# Clinicopathological Analysis of Factors Related to Colorectal Tumor Perforation: Influence of Angiogenesis

**DOI:** 10.1097/MD.0000000000000703

**Published:** 2015-04-17

**Authors:** Vicente Medina-Arana, Antonio Martínez-Riera, Luciano Delgado-Plasencia, Diana Rodríguez-González, Alberto Bravo-Gutiérrez, Hugo Álvarez-Argüelles, Antonio Alarcó-Hernández, Eduardo Salido-Ruiz, Antonia M. Fernández-Peralta, Juan J. González-Aguilera

**Affiliations:** From the Department of General and Digestive Surgery (VMA, LDP, DRG, ABG, AAH); Department of Internal Medicine (AMR); Department of Pathology, Hospital Universitario de Canarias, La Laguna, Tenerife (HAA, ESR); and Department of Biology-Genetics. Universidad Autónoma de Madrid, Madrid, Spain (AMFP, JJGA).

## Abstract

Colorectal tumor perforation is a life-threatening complication of this disease. However, little is known about the anatomopathological factors or pathophysiologic mechanisms involved.

Pathological and immunohistochemical analysis of factors related with tumoral neo-angiogenesis, which could influence tumor perforation are assessed in this study. A retrospective study of patients with perforated colon tumors (Group P) and T4a nonperforated (controls) was conducted between 2001 and 2010. Histological variables (differentiation, vascular invasion, and location) and immunohistochemical (CD31, Growth Endothelial Vascular Factor (VEGF) and p53) related with tumor angiogenesis were analyzed.

Of 2189 patients, 100 (4.56%) met the inclusion criteria. Of these, 49 patients had nonperforated (2.23%) and 51 had perforated tumors (2.32%). The P group had lower number of right-sided tumors (7/51, 13.7%) compared with controls (13/49, 36.7%) (*P* = .01). The high-grade tumors (undifferentiated) represented only 3.9% of the perforated tumors; the remaining 96.1% were well differentiated (*P* = .01). No differences between groups in the frequency of *TP53* mutation or *VEGF* and *CD31* expression were found. In the P group, only 2 (3.9%) had vascular invasion (*P* = .01). Of the 12 tumors with vascular invasion, only 2 were perforated (16.6%). The median number of metastatic lymph-nodes in P Group was 0 versus 3 in controls (Z = −4.2; *P* < .01).

Pathological analysis of variables that indirectly measure the presence of tumor angiogenesis (differentiation, vascular invasion, and the number of metastatic lymph nodes) shows a relationship between this and the perforation, location, and tumor differentiation. We could not directly validate our hypothesis, by immunohistochemistry of TP53, VEGF, and CD31, that perforated tumors exhibit less angiogenesis.

## INTRODUCTION

Colorectal cancer (CRC) is the second most common tumor in the Western world and the leading cause of cancer mortality. Obstruction and perforation are the most serious complications of the disease and occur in 2.6% to 10% of all patients with CRC.^1–4^ Although perforation is generally associated with a poor prognosis and high mortality rates, it has been suggested that this poor outcome is limited to cases with immediate postoperative complications of sepsis. Our group recently reported^5^ that patients with perforated colorectal tumors show better survival, related to better nodal stage, than patients with tumors that also reach the serosa but are not perforated. However, we know little about the pathophysiological factors or mechanisms underlying tumor invasion of the serosa ending with perforation in the abdominal cavity. As tumor growth requires rapid vascular supply, and the anti-angiogenic agents used in chemotherapy can cause perforation of the colon, we hypothesized that impaired tumor angiogenesis could be a factor that determines the perforation of these tumors.

The aim of the present work was to analyze a series of pathological and immunohistochemical factors related with tumor neoangiogenesis which, at least from a theoretical point of view, could influence the tumor perforation in CRC.

## MATERIALS AND METHODS

Participants were patients with colorectal adenocarcinoma, recruited between January 2001 and April 2010, treated with curative intent at the Department of Surgery, Hospital Universitario de Canarias (Tenerife) Spain. Patients were excluded if the operation was performed at an outside hospital, the tumor was in the middle or lower third of the rectum, or if patients had received preoperative radiotherapy and/or chemotherapy. In patients with metastasis at diagnosis, resection R0 of the primary tumor was performed first. Then the metastasis was treated with adjuvant chemotherapy and finally, when possible, resection was performed. T4 tumor patients showing histological serosal invasion were divided

into 2 groups: controls (patients with nonperforated tumors) and Group P (patients with perforated tumors). In Group P, tumors were spread across the surface of the visceral peritoneum (T4a) and had undergone spontaneous perforation. Only those with spontaneous perforation and complete penetration of the colon wall resulting in peritonitis were included. Iatrogenic tumor perforation during surgery or perforations in remote sites secondary to obstruction were excluded.^1^ Tumor perforation was identified by the surgeon during emergency surgery because the clinical profile of acute abdomen observed.

The following variables were collected: demographics (patient's sex and age at onset), tumor location (right, left, or at the junction of the rectum and sigmoid colon), pathological data (histological type, presence or absence of mucin, tumor differentiation analyzed by 2 different systems: the AJCC classification as high/low grade of differentiation, and a second system in which tumors were classified as undifferentiated, moderately and well differentiated), presence of metastatic lymph nodes and vascular invasion. We also analyzed factors related to angiogenesis (CD31 expression, mutated *TP53* and Growth Endothelial Vascular Factor (VEGF) in tumor tissue) using semi quantitative assessment by immunohistochemistry.

The study was reviewed and approved by the Research Ethics Committee, Hospital Universitario de Canarias (Code: 2012/26), on June 28, 2012.

### Immunohistochemistry

Immunohistochemistry was performed on formalin-fixed paraffin-embedded sections of tumor, after antigen retrieval in high pH solution (Dako), at 123_C for 1 min. Sections were treated with 0.1% Triton X-100 in phosphate buffered 170 saline (PBS), and then incubated with mouse monoclonal antibody, diluted 1:50 in 3% bovine serum albumin/PBS, against human VEGF human AB 1785 (Calbiochem Lab), mouse 172 anti-p53 mouse DO-7 anti-p53 (Dako Lab) and mouse anti CD31 mouse JC-70 A (Dako Lab). After blocking endogenous peroxidase with 0.3% H_2_O_2_ in methanol, for 15 min., slides were washed 3 times, 5 min each, with PBS and then incubated for 30 min with Horseradich Peroxidase-conjugated anti-mouse IgG serum (Dako). After another 3 PBS washes, a DAB-H2O2 solution was used as chromogen, and sections were counterstained with hematoxylin. Adjacent normal tissue and surrounding tissue lymphocytes served as internal positive controls for each case. Nuclear staining of the tumor was scored as either present or absent compared with the corresponding internal control. Tumors were classified into high (positive staining for vascular endothelial growth factor—VEGF—in 50% or more of cells) and low (positive staining for VEGF in 50% or fewer cells) VEGF expression,^6^ no expression (less than 10% positive tumor cells), and positive expression (more than 10% staining tumor cells) for p53^7,8^ and the presence of small and large vessel invasion for CD31.^9^

### Statistical Analysis

Perforated and nonperforated tumors were compared. Continuous variables were compared using Student *t* test or Mann–Whitney *U* test. Categorical variables were compared using chi-square test. *P* values ≤0.05 were considered significant. All statistical calculations were performed using the Statistical Package for Social Sciences (SPSS, version 15.0).

## RESULTS

**FIGURE 1:**
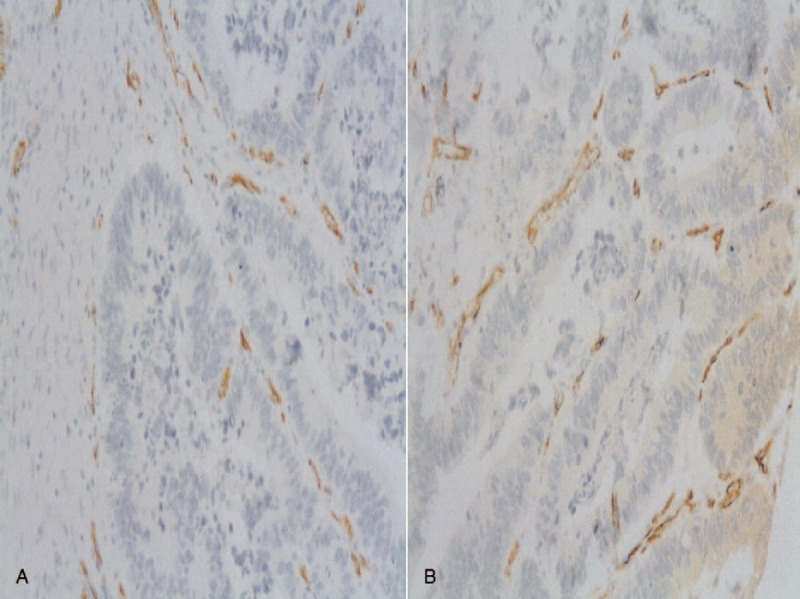
Inmunohistochemical image of CD31 in control group (A) and group P (B) (CD31 ×200).

**FIGURE 2:**
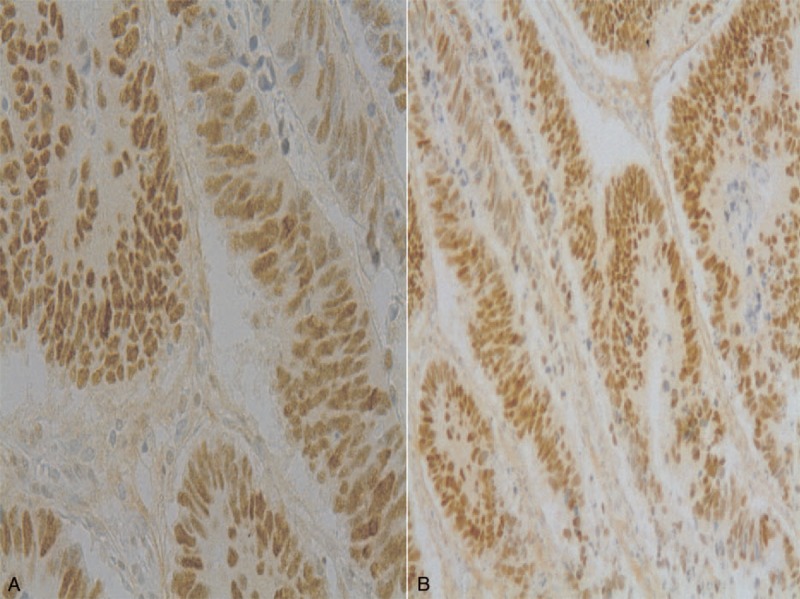
Inmunohistochemical image of p53 in control group (A) and group P (B) (P53 ×200).

**FIGURE 3:**
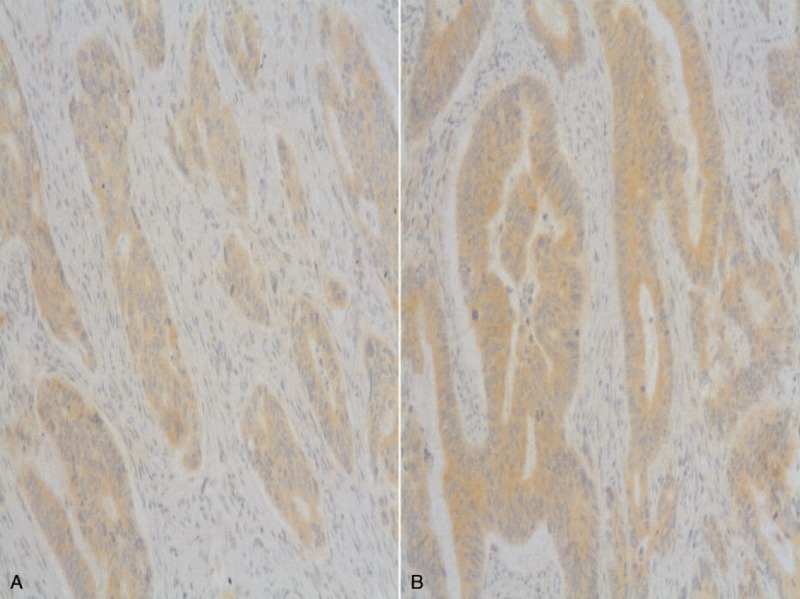
Inmunohistochemical image of VEGF in control group (A) and group P (B) (VEGF ×200).

Hundred out of 2189 patients (4.56%) met the inclusion criteria for the study. Forty-nine patients had a nonperforated tumor (2.23%) and 51 had a perforated tumor (2.32%). Table 1 shows the demographic characteristics (age and sex) of the 2 groups. No significant differences were found between groups for age, or age-group ≤50 years (recommended age for starting surveillance studies). For these variables, no significant differences between groups were observed. Table 2 shows the factors involved in tumor perforation. Firstly, regarding location, perforated tumors showed significantly fewer right-sided tumors (28%) than controls (72.0%) (*P* < 0.01). A high proportion of perforated tumors were located in the left colon and rectosigmoid junction (58.7%; *P* = .01). Cell differentiation also showed significant differences. High-grade tumors (undifferentiated) only accounted for 16.7% of the perforated tumors, only 2 of 12 (*P* = .01), whereas the remaining 83.3% were low-grade tumors (well differentiated). We found no relation of perforation and histological type (*P* = 0.44). Regarding tumor angiogenesis, perforated tumors tended to express more frequently (56.3%) the epithelial marker CD31 than controls (43.7%; *P* = 0.1) (Table 2). We did not found significant differences between groups in the frequency of *TP53* mutation, or VEGF expression (Table 2 and Figures 1–3). We also observed that tumors invading the serosa but are not perforated tumors showed frequently vascular invasion (*P* = .011) and a greater number of metastatic lymph nodes (*P* < 0.01) (Table 3).

**TABLE 1:**
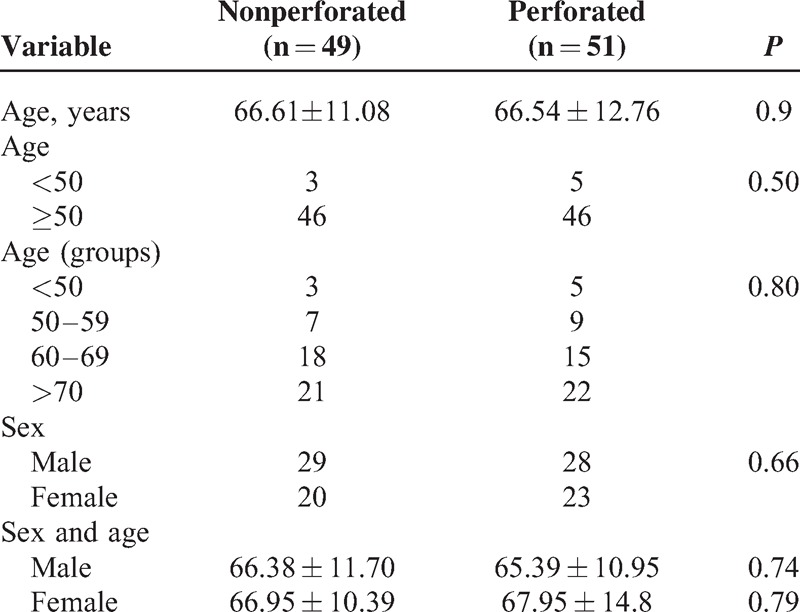
Demographic Characteristics of Patients With Nonperforated and Perforated T4a Tumors

**TABLE 2:**
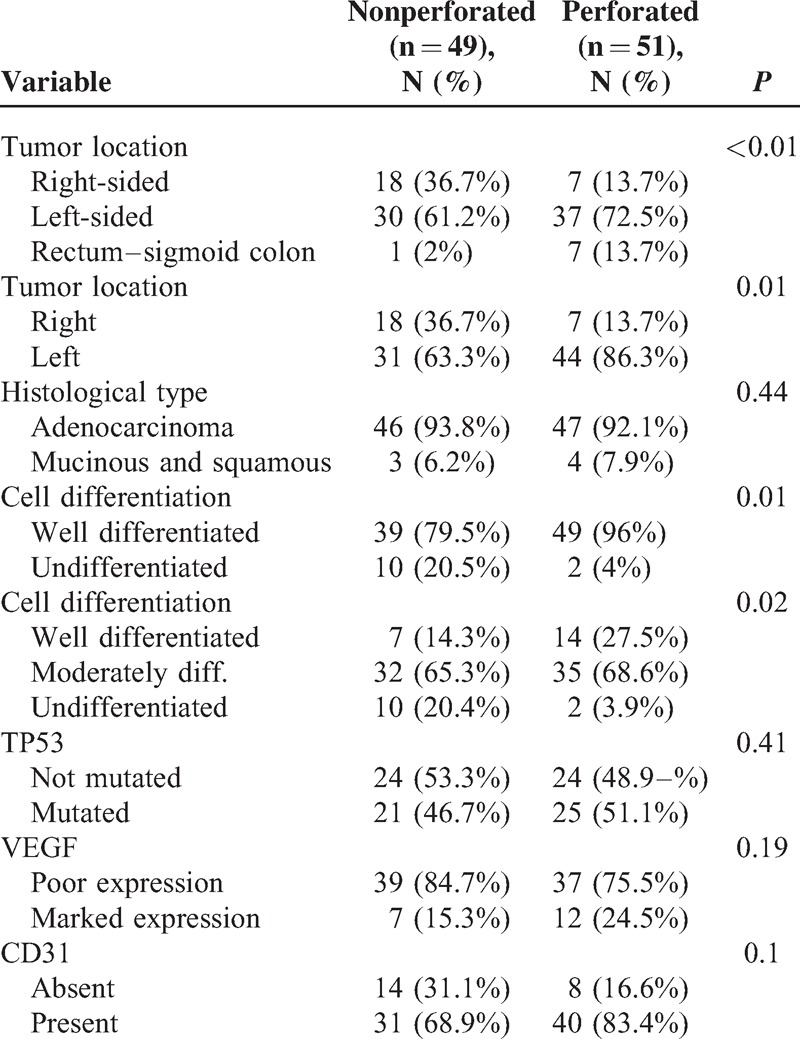
Factors Associated With T4a Tumors Perforation

**TABLE 3:**
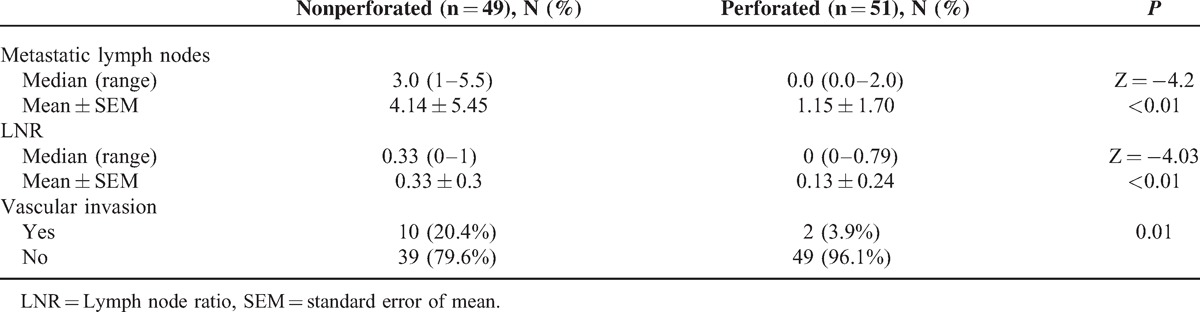
Metastatic Lymph Nodes and Vascular Invasion in T4a Nonperforated and Perforated Tumors

We also analyzed the possible effect of tumor differentiation in several variables independently that tumor was perforated or not (Table 4); in both perforated and nonperforated tumors, we found statistically significant differences in the location of the tumor in respect to level of differentiation. Thirty-two percent of undifferentiated tumors were right-sided compared with only 16% of well-differentiated tumors (*P* < 0.01). Moderate and undifferentiated tumors showing vascular invasion in 58.3% and 41% of cases, respectively, whereas all cases of well differentiated tumors showed no vascular invasion (*P* < 0.01). In addition, the median number of metastatic lymph nodes associated with undifferentiated tumors was 4 versus 0 for the well differentiated tumors (*P* = .02), and the median of lymph node ratio (LNR) was 0.3 versus 0 (*P* = .02) respectively. Undifferentiated tumors show expression of CD31 less frequently (45.4%) than the differentiated ones (83.3%; *P* = .03). Finally, we found no differences in either *TP53* mutation or VEGF in relation to the level of differentiation of the tumors.

**TABLE 4:**
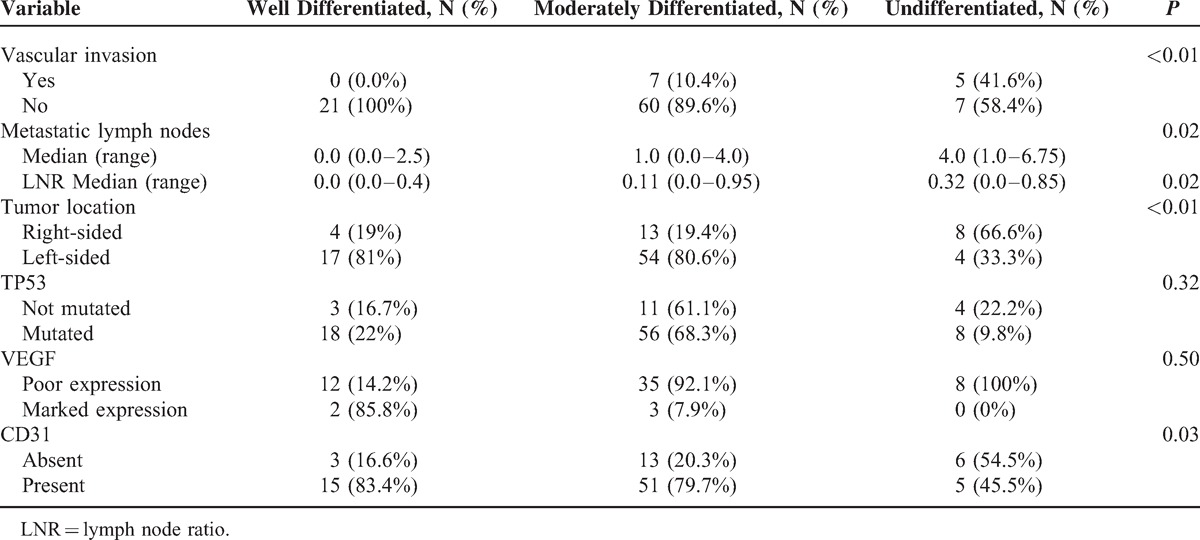
Interaction Between Degree of Cell Differentiation and Other Variables

## DISCUSSION

As tumor perforation has traditionally been regarded as a serious complication of colorectal tumors, analysis of the factors involved in perforation is of great importance and may shed light on the pathogenesis of tumor development. The only previous research we have found about factors associated with tumor perforation reported the association of the female sex with tumor perforation.^10,11^ But the anatomopathological data and location of tumors were not analyzed. We have not found such relation neither with sex, nor patient age (Table 1).

Differences in the frequency of tumor perforation in relation to location was found, those located on the left side (*P* = .01), mainly at the rectosigmoid junction (*P* < .01) (Table 2), were more frequently perforated than right-sided tumors. This finding may be explained by different characteristics between right and left-sided tumors.^12^ Proximal or right-sided tumors usually present as exophytic polypoid masses, whereas distal or left-sided tumors are usually annular lesions that produce constriction and narrowing of the intestinal segment. In addition, during embryonic development, the peritoneum is capable of binding, at a certain stage of development, the arrangement of the organs by a process called coalescence so that some floating portions of the gastrointestinal tract become fixed to the wall by partial or total coalescence of their meso with the posterior parietal peritoneum. Coalescence occurs along all the colon, but stops at the level of the sigmoid colon, from the innominate line to the third sacral vertebra, so this portion of the colon up to the rectosigmoid junction is not bound and retains its mobility, with both peritoneal sheets forming the intersigmoid recess.^13^ This defect of peritoneal coalescence can promote perforation of this portion more frequently than in the remaining where the complete coalescence occurs, forming the fascia that acts as a barrier to perforation.

Tumor cell differentiation also influences perforation; in tumors with a low degree of differentiation (well differentiated), perforation was more frequent, whereas highly differentiated (undifferentiated) tumors tended to progress and not become perforate (*P* = 0.01) (Table 2). This may lead to that these last tumors show an increase in vascular invasion (*P* = 0.01) (Table 3) and a greater number of metastatic lymph nodes (Z = −4.2, *P* < 0.01) and LNR (Z = −4.03, P < 0.01) (Table 3), which will eventually lead to worse survival, as reported previously by our group.^5^

When a tumor forms, its initial development is characterized by an avascular phase with no more than 2 to 3 mm of growth.^3^ Then a change to a vascular phase occurs, known as angiogenesis switch. A cascade of events results in volume expansion and subsequent metastasis. The formation of new vessels is the mechanism by which tumor lesions in situ, avoid the critical limitations of oxygen diffusion and nutritional constraints. Angiogenesis is, therefore, an essential process for the growth and progression of solid tumors in general. Although there are many growth factors that regulate angiogenesis, VEGF is the most widely studied and perhaps the most important. Its activation stimulates the growth, migration, and survival of vascular endothelial cells. In in vitro experiments, the expression of an anti-apoptotic protein in endothelial cells (Bcl-2) is also stimulated.^14^ VEGF is expressed in colorectal cancer, and its expression correlates with tumor progression and poor prognosis.^7^ That is why anti-angiogenic agents for the treatment of colorectal carcinoma have been developed. These agents have as the most relevant side-effects, although infrequently, increased bleeding and perforation of the colon.^15^ A major inhibitor of angiogenesis is the suppressor gene *TP53*. This gene stops cell cycle progression in nonviable conditions by inducing hypoxia-mediated apoptosis.^7^*TP53* is mutated in 50% of all cancers, including colorectal cancer. The function of *TP53* is mediated through the proteins it produces, which stop cells in the G1 phase of the cell cycle or induce apoptosis on detecting DNA damage.^16^

Given the above, we have attempted to assess angiogenesis by semiquantitative measurement of VEGF and p53, but have found no relationship between the 2 and the presence or absence of perforation (Table 2). Rajest et al^17^ studied the correlation between VEGF and CD31 in breast cancer cells and found no correlation between these 2 markers; they concluded that multiple angiogenic factors must play a role together with VEGF in the angiogenic process. We also analyzed CD31 to try to demonstrate the presence of endothelial cells in tumor tissue sections, as this is used to assess the degree of tumor angiogenesis and may be implicated in rapid tumor growth. We observed a trend toward increased expression of CD31 in perforated tumors, that is, increased angiogenesis (Table 2). Zółtowska et al^18^ report that tumoral cells of lung, breast, and colorectal cancer not always show CD31 expression in capillaries, especially in tumors with low differentiation where the vessels are discontinuous or with interruptions. In our study, undifferentiated tumors were the least perforated (Table 4), and yet had a lower uptake of CD31 probably because the neovessels were discontinuous or interrupted as described above.^19^

Histological grade reflects the level of tumor differentiation and it has proved to be an independent prognostic factor.^4^ The AJCC/UICC recommends applying a system of 2 degrees in relation to the number of glands.^20,21^ The tumor cell differentiation determines the degree of tumor angiogenesis necessary for growth. The greater the degree of differentiation (well differentiated) the less the level of angiogenesis^6,22^ and this leads to greater hypoxia and necrosis, probably because *TP53*, which induces cell apoptosis, is maintained intact. The consequence of these phenomena is less vascular and lymphatic invasion which determines less regional lymph node metastases. Well-differentiated tumors in this situation would be associated with reduced angiogenesis and increased hypoxia and necrosis, resulting in a higher rate of perforation.

Vascular invasion refers to the invasion of the tumor into the veins or small vessels without muscle cells, which represent venules or postcapillary lymphatic that is an independent adverse prognostic factor, just as lymph node invasion is. It has been described a relationship between angiogenesis and vascular invasion.^6,14^ We have observed that 2 of 12 tumors with vascular invasion showed perforation 16.7%, whereas 49 of 88 tumors without vascular invasion showed perforation (55.7%). The absence of vascular invasion is an indirect sign of a low rate of angiogenesis, which means less oxygen supply to tumor tissue and greater likelihood of perforation. Furthermore, this fact leads to a smaller presence of tumor cells in the vessels and a lower number of lymph node metastases (Table 3).

**FIGURE 4:**
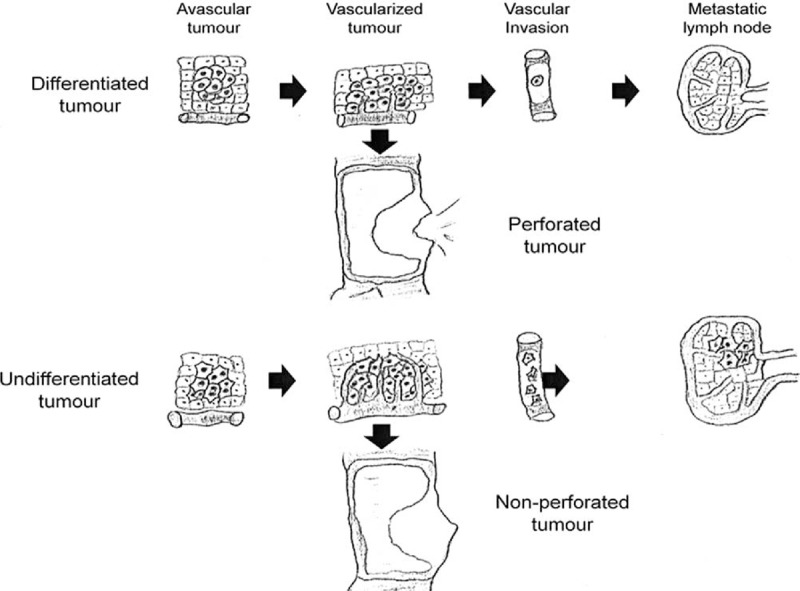
Possible mechanism of interaction between the degree of tumor differentiation, vascular invasion, angiogenesis, and lymph node invasion in perforated and nonperforated tumors.

Finally, we analyzed the relationship between degree of cell differentiation in the tumor (well, moderately, or undifferentiated) and other variables (Table 4). Well differentiated tumors were predominantly left-sided (17 of 21), showed no vascular invasion (21 of 21), and had a median number of metastatic lymph of 0, in contrast to undifferentiated tumors, which were predominantly right-sided (8 of 12), evidenced vascular invasion (5 of 12), and had a median number of metastatic lymph nodes of 4. All these differences were highly statistically significant and demonstrate a relationship between differentiation, vascular invasion, and nodal involvement. Our results suggest that the 3 variables analyzed can be used as markers of tumor angiogenesis, as shown depicted in Figure 4.

One of the questions that arises, if our theory is correct, is how to explain the pathogenesis of early stage tumors. For example, unperforated tumors at stage T2 or T3 may have less angiogenesis preventing their growth and make them, at least in theory, more susceptible to perforation. However, this lack of angiogenesis would likely produce tumor necrosis but not perforation, as the tumor does not extend to all the layers of the colonic wall.

In conclusion, this study shows a relationship between tumor angiogenesis and perforation by analyzing different pathological variables that indirectly measure the presence of angiogenesis (differentiation, vascular invasion, and the number of metastatic lymph nodes). Although we could not directly validate our hypothesis that perforated tumors exhibit less angiogenesis by immunohistochemical analysis of TP53, VEGF, and CD31. Tumor neoangiogenesis is a very complex process involving more elements^23^ than those considered here. For example, the results obtained by Barbera-Guillem et al^24^ suggest that B-lymphocytes (CD19 and CD21-labeled) can enhance tumor progression by inducing neoangiogenesis in tumor stromal.

The main limitation of this study is that it analyzes angiogenesis by indirect methods, so immunohistochemical analysis of the 3 variables studied may be insufficient even when analyzed together. Other more direct methods, such as VEGF determinations of blood or the use of new markers of angiogenesis in endothelial cells and CD105 expressed on the endothelium of carcinoma cells and underexpressed in normal endothelial cells,^25^ could help to elucidate the role of angiogenesis in perforated colon cancer. Further studies are required to demonstrate a direct relationship between tumor angiogenesis and perforation.
